# Autopsy histology data suggest cirrhosis is frequently under-reported on death certificates

**DOI:** 10.1097/HC9.0000000000000015

**Published:** 2023-01-18

**Authors:** Hannes Hagström, Tracey G. Simon, Jonas Söderling, Jonas F. Ludvigsson

**Affiliations:** 1 Division of Hepatology, Department of Upper GI, Karolinska University Hospital, Stockholm, Sweden; 2 Department of Medicine, Huddinge, Karolinska Institutet, Stockholm, Sweden; 3 Department of Medical Epidemiology and Biostatistics, Karolinska Institutet, Stockholm, Sweden; 4 Clinical Epidemiology Unit, Department of Medicine, Solna, Karolinska Institutet, Stockholm, Sweden; 5 Department of Pediatrics, Örebro University Hospital, Örebro, Sweden; 6 Department of Medicine, Columbia University College of Physicians and Surgeons, New York, New York, USA

## Abstract

Cirrhosis may be asymptomatic yet contribute to ill health. Here, we studied how often cirrhosis is discovered first at an autopsy. We also examined how frequent this finding was not reported on formal death certificates.

**Methods:**

We used register-based data from all liver biopsies in Sweden performed after death. Cross-linkage to national registers was performed to examine how often such patients had accurate coding for cirrhosis on their death certificates.

**Findings:**

A total of 6187 patients with a liver biopsy performed after death, showing cirrhosis. Of these, 2523 (41%) did not have a diagnosis corresponding to cirrhosis on their final death certificate.

## INTRODUCTION

Chronic liver diseases are common and can lead to cirrhosis. Cirrhosis is frequently asymptomatic and is often diagnosed at a late stage.^1^ However, cirrhosis is associated with tremendous morbidity, mortality, and impaired quality of life.^2^–^4^ Thus, accurate reporting of cirrhosis in healthcare registers is important to track changes that can guide health interventions. However, such data relies on the correct diagnosis of cirrhosis on reports such as death certificates. It is not known how common undiagnosed cirrhosis might be at death, or how frequently it is correctly reported as a cause of death, on death certificates. Downstream consequences of cirrhosis, such as infections or fractures due to sarcopenia, etc., might not be accurately identified as such when physicians fail to register cirrhosis. Here, we aimed to estimate how often cirrhosis is found on autopsy reports and compare this to data from official death certificates. The study was approved by the Stockholm Ethics Review Board (No. 2014/1287-31/4). Because this is a register-based study using anonymized data and no patient contact, the Ethics Review Board waived informed consent.

We identified 42,495 patients with a liver biopsy performed after death using validated Swedish registers.^5^–^7^. Of these, 6187 had cirrhosis as defined by biopsy. Of these, 2523 (41%) did not have a diagnosis corresponding to cirrhosis on their death certificate (Table 1). Patients without a diagnosis of cirrhosis on their death certificate were older compared to patients where this was not reported (median 73 vs. 67 years, *p*<0.001), but there was no difference in sex (65% males vs. 63%, *p*=0.24). The test characteristics of a diagnosis of cirrhosis in the causes of death register, using data from the liver biopsy as gold standard, were: sensitivity=59.2%, specificity=94.1%, positive predictive value=63.0%, negative predictive value=93.1%.

**TABLE 1:** Characteristics of patients with a code for cirrhosis on autopsy-based liver biopsy, and those with also a code for cirrhosis on the death certificate

Characteristic	Cirrhosis on autopsy (n=6187)	Cirrhosis also on death certificate (n=3,664)	Cirrhosis not on death certificate (n=2523)	*p* a
Sex, n (%)				
Women	2233 (36.1)	1344 (36.7)	889 (35.2)	0.24
Men	3954 (63.9)	2320 (63.3)	1634 (64.8)	
Age at date of death (y)				
Mean (SD)	68.4 (13.3)	66.3 (12.9)	71.4 (13.2)	<0.001
Median (IQR)	69.6 (60.0–78.1)	67.1 (57.8–76.0)	73.4 (64.7–80.7)	<0.001
Range, minimum–maximum	0.2–97.2	0.2–96.4	0.3–97.2	
Categories, n (%)				
<18 y	35 (0.6)	17 (0.5)	18 (0.7)	<0.001
18–<50 y	483 (7.8)	362 (9.9)	121 (4.8)	
50–<65 y	1754 (28.3)	1242 (33.9)	512 (20.3)	
≥65 y	3915 (63.3)	2043 (55.8)	1872 (74.2)	
Country of birth, n (%)				
Nordic country	5921 (95.7)	3501 (95.6)	2420 (95.9)	0.49
Other	266 (4.3)	163 (4.4)	103 (4.1)	
Year of death				
1969–1989	924 (14.9)	572 (15.6)	352 (14.0)	0.11
1990–2000	2805 (45.3)	1622 (44.3)	1183 (46.9)	
2001–2010	1854 (30.0)	1118 (30.5)	736 (29.2)	
2011–2017	604 (9.8)	352 (9.6)	252 (10.0)	
Underlying liver disease ever before death, n (%)^b^				
Viral hepatitis	535 (8.6)	384 (10.5)	151 (6.0)	<0.001
Alcohol-related liver disease	1723 (27.8)	1439 (39.3)	284 (11.3)	<0.001
NAFLD	63 (1.0)	50 (1.4)	13 (0.5)	0.001
Autoimmune liver disease	199 (3.2)	141 (3.8)	58 (2.3)	<0.001
Other liver diseases	96 (1.6)	66 (1.8)	30 (1.2)	0.056
Cirrhosis	2711 (43.8)	2287 (62.4)	424 (16.8)	<0.001
Decompensated cirrhosis	1633 (26.4)	1427 (38.9)	206 (8.2)	<0.001
Any known liver disease	3470 (56.1)	2750 (75.1)	720 (28.5)	<0.001
No known liver disease	2717 (43.9)	914 (24.9)	1803 (71.5)	
Most commonly reported underlying causes of death				
Digestive including liver	2355 (38.1)	2088 (57.0)	267 (10.6)	<0.001
Cardiovascular	1986 (32.1)	744 (20.3)	1242 (49.2)	
Tumors	873 (14.1)	389 (10.6)	484 (19.2)	
Respiratory	265 (4.3)	115 (3.1)	150 (5.9)	
Infections	171 (2.8)	119 (3.2)	52 (2.1)	
Endocrine	146 (2.4)	71 (1.9)	75 (3.0)	
Genitourinary	66 (1.1)	30 (0.8)	36 (1.4)	
Psychiatric	60 (1.0)	23 (0.6)	37 (1.5)	
Other	265 (4.3)	85 (2.3)	180 (7.1)	

^a^ χ^2^ test, Student *t* test, or Wilcoxon rank-sum test as appropriate. Comparing patients with/without cirrhosis on the death certificate.

^b^ Patients could have more than 1 reported liver disease, for example, both viral hepatitis and cirrhosis. Figures represent the number of patients with a recorded diagnosis of liver disease in the National Patient Register prior to death. For example, 39.3% of patients with cirrhosis registered on the death certificate had an earlier diagnosis of alcohol-related liver disease. This compares with 11.3% of those whose cirrhosis was not registered on the death certificate.

Abbreviations: IQR, interquartile range.

In total, 2717 patients (43.9%) of patients with biopsy-confirmed cirrhosis did not have a known liver disease, such as alcohol-related liver disease, recorded in the National Patient Register at any time prior to death.

Undiagnosed liver disease was more common in patients without cirrhosis recorded on the death certificate (n=1803, 71.5%), compared to those where cirrhosis was also reported on the death certificate (n=914, 24.9%, *p*<0.001). That is, in patients where liver disease was known prior to death it was more commonly recorded on the death certificate.

Further, we found that the proportion of patients with unrecorded cirrhosis was stable over time (42% in the 2011–2017 period), but that the absolute number of autopsies declined in the later decades (Table 1), in accordance with previous studies.^8^ In those without cirrhosis noted on the death certificate, the most commonly reported causes of death were cardiovascular diseases (49%) or tumors (19%).

Thus, we show that in patients with autopsy-confirmed cirrhosis, over 40% do not have mention of cirrhosis on the final death certificate. In most of those where cirrhosis was not reported on the death certificate (72%), no known liver disease was known prior to the autopsy, whereas known liver disease was found in the majority of those where cirrhosis was reported on death certificate (75%). This signals a problem with the sensitivity of death certificate reports to accurately classify presence of cirrhosis. A plausible explanation is that updated death certificates (which are not mandatory) were not submitted by the clinician receiving the pathologists report with the information of cirrhosis. Another explanation could be that the cause of death was considered completely unrelated to cirrhosis, although this seems unlikely based on the top 5 causes of death identified in the examined groups since cirrhosis is highly associated with such diseases. We also cannot rule out selection bias, for instance patients that die due to known cirrhosis might not have been subjected to autopsy.

These novel results highlight that even in a highly organized country such as Sweden with extensive registers, cirrhosis is frequently under-reported on death certificates, and the autopsy often the first time point where cirrhosis is recorded. In spite of acknowledgment of cirrhosis at the autopsy, the death certificate frequently fails to capture this. This is problematic since such reports form the basis of much epidemiological research on disease trends, and the proportion of deaths where autopsies are performed is declining.^8^ Recent studies show an increase in the mortality of liver diseases globally.^9^ Coupled with a marked reduction in the number of autopsies performed, this figure could be understated, and cirrhosis might be a more important contributor to death than previously thought.
